# Nursing Sore Mouth

**Published:** 1869-11

**Authors:** 


Nursing Sore Mouth.—This troublesome affection is spoken of in these terms by Dr. I. P. Wilson, in the *St. Louis Medical Journal.* Dr. Wilson is of opinion that it is the result of an impoverished condition of the system.

The child in embryo and in infancy is supported by its mother. The mother's system is continually being drained from the day of conception to the time she weans her child. She has not only her own body to maintain during gestation and lactation, but her offspring must be supplied with the bone, muscle, and nerve producing materials, even though her own system be starved for the purpose. If the system is robbed of any of its constituent parts, the body must suffer. The bones, e. g. contain from 48 to 59 per cent. of the phosphate of lime, and the enamel of the teeth from 81 to 88 per cent., hence an immense supply of these lime salts is required to maintain the mother, and to build up the bony tissues of the child. Stomatitis materna is nearly always accompanied by extreme sensitiveness of the teeth, and a softening of the tooth structure, showing a starved condition of the entire osseous system. The lime salts have been appropriated for the development of the bony tissues of the child, while the exhausted mother is suffering the consequences of an impoverished system.

This disease is more prevalent with pregnant and nursing females, because they demand a far greater supply of those life-supporting elements; but it is not this class alone that suffers from this condition The non-pregnant female who is living on a poor, weak diet, is liable to suffer the same consequences. The male sex, too, may have sore mouth of the same character, but it is always given some other name, and attributed to some other cause.

In my practice as a dental physician I have been called upon to treat this disease, and when it has not progressed too far, I have only found it necessary to recommend a good, nutritious diet, with plenty of exercise in the fresh air and in the sun. If the entire alimentary canal is affected, tonics should be given, and a general constitutional treatment may be required.

One or two kinds of aliment will not keep the system in repair. A variety is necessary. Milk and eggs are said to be the only articles of food that contain all the required elements. The lime salts abound richly in the unbolted wheat flour, while fine flour is almost entirely destitute of this element.

Let the mother's system be furnished with a sufficient amount of the bone, muscle and nerve producing materials to build up the tissues of her child, in utero and during infancy, and "stomatitis materna" will rarely if ever exist.

